# Improvement of sports nutrition knowledge by a dual method education program in track and field athletes: a randomized controlled trial

**DOI:** 10.1080/15502783.2025.2519517

**Published:** 2025-06-17

**Authors:** Ranil Jayawardena, Kalani Weerasinghe, Indu Nanayakkara, Terrence Madhujith, Andrew P. Hills, Nishan Sudheera Kalupahana

**Affiliations:** a University of Colombo, Department of Physiology, Faculty of Medicine, Colombo, Sri Lanka; b University of Peradeniya, Department of Physiology, Faculty of Medicine, Kandy, Sri Lanka; c University of Colombo, Health and Wellness Unit, Faculty of Medicine, Colombo, Sri Lanka; d University of Peradeniya, Department of Food Science and Technology, Faculty of Agriculture, Kandy, Sri Lanka; e University of Tasmania, School of Health Sciences, College of Health and Medicine, Launceston, Australia; f United Arab Emirates University, Department of Nutrition and Health, College of Medicine and Health Sciences, Al Ain, United Arab Emirates

**Keywords:** Sports nutrition knowledge, nutrition education, track and field, athletes, Sri Lanka

## Abstract

**Background:**

Athletes with strong sports nutrition knowledge are believed to have sound nutritional practices and better performance. This study aimed to evaluate the effectiveness of a dual-method personalized nutrition education program in improving sports nutrition knowledge (SNK) among Sri Lankan track and field athletes.

**Methods:**

This parallel-group, randomized controlled trial recruited national-level track and field athletes aged 18 and above. The intervention group (IG) received personalized sports nutrition consultations at 0, 4^th^, and 8^th^ weeks, along with online educational materials via WhatsApp from weeks 10 to 16, while the control group (CG) did not receive any intervention. A per-protocol analysis was followed, and t-tests were used to compare the means.

**Results:**

Of the 30 participants enrolled, 13 from IG and 14 from CG completed the study. Following the 16-week intervention, the IG demonstrated significant improvements in total nutrition knowledge (TNK) relative to the CG (IG: 34.41 ± 4.15 vs. CG: 20.96 ± 4.38; *p* = 0.01), with significant increases in general nutrition knowledge (GNK) (IG:24.54 ± 3.66 vs. CG:15.64 ± 2.33; *p* = 0.004) and SNK (9.87 ± 3.87 vs. 5.32 ± 4.07; *p* = 0.006). Changes in TNK were also significantly greater in the IG compared to the CG; (IG:9.45 ± 0.10 vs. CG: −2.63 ± 0.37; *p* < 0.0001), GNK (IG:4.47 ± 0.08 vs. CG: −1.28 ± 0.09; *p* < 0.0001), and SNK (IG:4.99 ± 0.72 vs. CG: −1.25 ± 0.74; *p* < 0.0001).

**Conclusions:**

The 16-week dual-method nutrition education intervention was effective in improving TNK, GNK, and SNK among track and field athletes in Sri Lanka.

**Trial registration:**

This trial is registered at the Sri Lanka Clinical Trials Registry (SLCTR/2024/013), Universal Trial Number (UTN): U1111–1304–8890 on 10 April 2024.

## 1. Introduction

Optimal nutrition is considered one of the fundamental pillars of peak physical condition, reducing fatigue, minimizing the risk of disease and injury, and general well-being in athletes [1]. Hence, appropriate nutrition through adequate intake of total energy, correct macronutrient composition, and sufficient micronutrients is essential for optimizing the performance of elite athletes [2]. Evidence indicates that carbohydrate intake is a vital fuel source for the performance of endurance athletes, as it helps maintain blood glucose levels during exercise and replenishes muscle glycogen through synthesis, ultimately leading to improved performance [3]. Sufficient protein intake is also necessary for tissue building and regeneration, while adequate fat intake provides essential fatty acids and fat-soluble vitamins [4]. Routine exercise may increase nutrient turnover, necessitating greater micronutrient intake to meet the increased needs for building, repairing, and maintaining lean body mass in athletes [5]. Despite the critical role of proper nutrition, previous studies have shown that many athletes do not meet the recommended energy, macronutrient, and micronutrient intakes [6].

A study among male and female athletes in Portugal from various sports revealed that nearly half reported inadequate carbohydrate intake [6]. During both the preparatory and competitive phases, the micronutrients with the most significant discrepancies between actual and recommended intakes were vitamins D and E, magnesium, folate, calcium, and zinc for both sexes and iron for females [6]. Additionally, female athletes are less likely to meet the recommended dietary fiber intake, possibly due to lower energy consumption compared to male counterparts [7]. In contrast, multiple studies have found that many athletes consume more total fat than the recommended amount [7,8].

Although the reasons for poor dietary practices among athletes are multifaceted, a lack of up-to-date, evidence-based sports nutrition knowledge (SNK) is a significant contributing factor [9]. Insufficient nutrition education is a primary reason for this lack of knowledge among athletes and coaches [9]. A systematic review among athletes and coaches concluded that athletes often have inadequate nutrition knowledge in areas such as energy density, micronutrients, supplementation, and weight management [10]. A cross-sectional study conducted among Iranian elite athletes demonstrated that their nutrition knowledge, attitude, and practices were below the expected levels [11]. However, findings across the literature are mixed. While some non-athlete groups, such as dietetics students, have scored higher than athletes, in other cases, elite athletes from developed, resource-rich countries like New Zealand have scored higher than non-athletic cohorts [10].

SNK, a key determinant of dietary behavior in athletes, should be evidence-based and culturally specific [12]. However, determining the current state of nutrition knowledge in athletes and coaches is challenging, as sports nutrition guidelines are continually updated, and different groups of athletes have varying nutritional requirements [10]. Therefore, well-validated sports nutrition knowledge questionnaires (SNKQs) are necessary to accurately assess the SNK of athletes and the practitioners who collaborate with them. In this context, we have developed evidence-based, culturally specific sports nutrition guidelines for track and field athletes in Sri Lanka and a well-validated SNKQ to assess their knowledge, known as the Sri Lankan-Sports Nutrition Knowledge Questionnaire (SLn-SNKQ) [13,14].

Previous research indicates that nutrition education interventions can positively impact athletes’ SNK. A randomized controlled trial found that swimmers increased their total SNK score by 8.3% (*p* = 0.006) after participating in a seven-week, interviewer-led nutrition education program [15]. Another study found that a validated booklet-based sports nutrition education intervention significantly increased knowledge scores (*p* < 0.001) among Malaysian team sports athletes, while the control group’s scores declined [16]. Further, an eight-week remote nutrition education program improved SNK scores by 15% (*p* < 0.001) among elite triathletes, concluding that such programs effectively enhance SNK and dietary intake, especially when face-to-face education is limited [17]. Heaney and colleagues reported that improved sports nutrition knowledge is associated with better dietary behaviors, particularly in achieving energy balance and aligning macronutrient intake with recommendations [18]. Similarly, another study highlighted that both athletes and coaches with higher nutrition knowledge are better equipped to make informed dietary decisions, which can enhance athletic performance and recovery [10]. However, the current understanding of SNK and dietary changes resulting from nutrition education interventions delivered through a combination of face-to-face meetings and online support for elite track and field athletes is limited. Therefore, this study aimed to evaluate the effectiveness of a 16-week, evidence-based, culturally appropriate nutrition education program, delivered through dual methods, in improving SNK among track and field athletes in Sri Lanka.

## 2. Methods

The CONSORT 2010 statement guidelines for randomized trials [19] were followed for this RCT, and the checklist is attached as Supplementary material 1 [20].

### 2.1. Trial design

**Figure 1.**
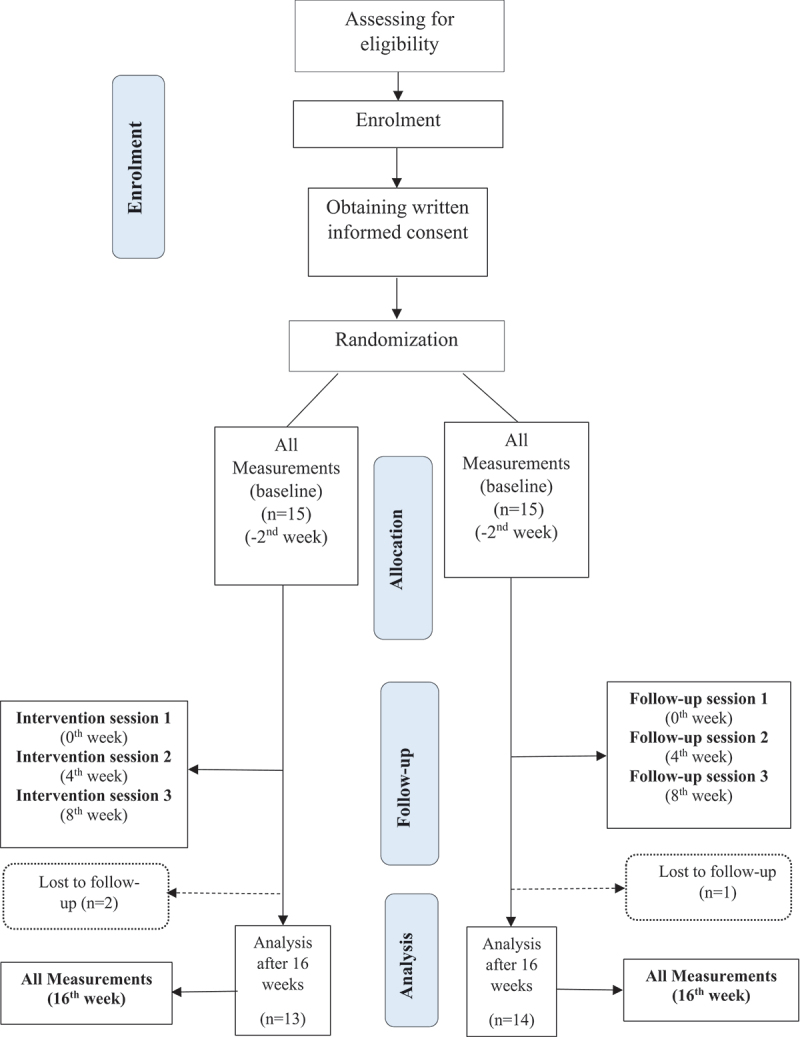
CONSORT diagram.

This study was designed as a parallel-group, randomized controlled clinical trial to evaluate the effect of dual education methods on SNK among elite athletes. The study was conducted at the Department of Physiology, Faculty of Medicine, University of Colombo, Sri Lanka. Written informed consent was obtained from all participants upon recruitment, and they were given the option to withdraw from the study or continue receiving support through regular follow-ups without any impact on their nutrition education. Ethical approval was granted by the Institutional Ethical Review Committee at the Faculty of Medicine, University of Peradeniya, Sri Lanka (2023/EC/71). This trial is registered with the Sri Lanka Clinical Trials Registry (2024/013), with a Universal Trial Number (UTN): U1111–1304–8890. The CONSORT diagram (Figure 1) illustrates the flow of this RCT.

### 2.2. Participants

Eligible participants included either elite or highly trained track and field athletes of both genders. In accordance with the participant classification framework proposed by McKay et al [21], highly trained athletes (Tier 3) are those competing at the national level with structured, periodized training and regular competition experience. Elite athletes (Tier 4) are defined as those competing at the highest international level (e.g. representing their country in major international competitions), with advanced training history and performance outcomes. Potential athletes were recruited through contacts with coaches, fellow athletes, and open advertisements in social media groups. Initial demographic information, including age, gender, years of athletic experience, and training regimen, was collected to ensure a representative sample of national-level athletes. Inclusion criteria were national-level athletes aged 18 years and above, both male and female, with a personal WhatsApp number (WhatsApp Inc., 1601 Willow Road, Menlo Park, California, United States of America), and full-time athletes willing to participate in the 16-week nutrition intervention. Athletes with current sports-related injuries or those receiving professional nutritional advice were excluded to ensure that participants did not have concurrent professional nutrition education during the study. While prior nutrition education was not directly assessed during the initial screening, the exclusion criteria helped minimize potential confounding from professional guidance. However, it was not possible to recruit participants with zero nutrition knowledge, so we expected to improve their knowledge due to the intervention, especially when compared to the control group. Potential athletes were recruited through contacts with coaches, fellow athletes, and open advertisements in social media groups. The intervention group (*n* = 15) participated in a sports nutrition education intervention through dual methods, while the control group (*n* = 15) was monitored throughout the study.

### 2.3. Intervention

The intervention comprised personalized sports nutrition consultations conducted by the principal investigator (RJ). It included three strategically spaced sessions delivered through dual methods. The first method consisted of three 15–30-minute individual in-person consultations at weeks 0, 4, and 8. The second method involved a series of online supports, where weekly sports nutrition education materials from national sports nutrition guidelines [22] were shared via WhatsApp from the 10^th^ to the 16^th^ week (Table 1). The development and validation of this structured, evidence-based, and culturally specific guideline are described in detail in a forthcoming publication [22], in which the complete version is provided as supplementary material.

**Table 1.** Sports nutrition education intervention outline.

Timepoints	Mode of delivery	Topics covered
Method I: Individual in-person consultations		
(1) 0^th^ week	15–30-min individual in-person face-to-face consultation	Fundamentals of macronutrients, fundamentals of micronutrients, energy balance, hydration
(2) 4^th^ week	15–30-min individual in-person face-to-face consultation	Weight management, sports supplements, training meals
(3) 8^th^ week	15–30-min individual in-person face-to-face consultation	Carbohydrate loading, doping, food habits while traveling, supplement label reading
Method II: Online sessions 10^th^ to 16^th^ week	Online; via WhatsApp	All the above-mentioned topics as digital materials

Different topics were discussed during the individual in-person consultations, as shown in Table 1. Throughout this period, the research team maintained regular communication with the athletes, addressing their queries, providing follow-ups, and offering ongoing support via WhatsApp.

The second part of the intervention, conducted online from the 10^th^ to the 16^th^ week, involved sharing a well-developed sports nutrition guideline – submitted elsewhere for publication – with ten chapters covering areas of both GNK and SNK. These guidelines were disseminated to the athletes weekly via WhatsApp, both as text messages and in PDF format in local languages (Sinhalese and Tamil) [22]. Athletes were instructed not to share the educational materials with anyone else before the conclusion of the intervention. They were also given ample opportunities to ask questions about content they were unclear about via call or text messages. Furthermore, participants were regularly followed up by a trained research assistant (KW), who asked random questions from each chapter weekly to ensure compliance with the intervention.

### 2.4. Control group

Participants in the control group did not receive any nutrition consultations or personalized interventions during the study period. However, they were advised to maintain their usual dietary patterns and refrain from seeking specialized nutrition advice or consuming supplements from other health or nutrition professionals to minimize confounding. In Sri Lanka, there are very few available professionals to provide nutritional advice, and the control group did not receive such guidance during the intervention. However, we ensured they were given nutritional advice after the intervention.

All participants, including those in the control group, were provided with general information on maintaining a balanced diet during the initial study briefing, ensuring they had access to standard nutritional knowledge. These measures aimed to ensure that any observed differences between groups could be attributed to the intervention. Participant adherence to these guidelines was monitored through direct communication via personal WhatsApp messages and periodic check-ins by the research team.

### 2.5. Outcomes

The primary outcome was the proportion of participants achieving a 10% or greater increase in mean SNK scores at the end of week 16 in the intervention group compared to the control group, as assessed using the validated SLn-SNKQ. The SLn-SNKQ measures both GNK and SNK across 12 subsections, with a maximum achievable score of 75. The focus on a 10% improvement threshold as the primary outcome was informed by prior research that demonstrated this level of change to be both achievable and meaningful in the context of sports nutrition education. Specifically, a randomized controlled trial reported a nearly 13% increase in total nutrition knowledge following a 12-week intervention among baseball players, which was also associated with significant improvements in nutrition-related behaviors and aspects of sports performance [23]. While the primary outcome focused on SNK due to its direct relevance to athletic performance, GNK improvements were also measured to provide a comprehensive evaluation. The SLn-SNKQ was meticulously developed and validated through a rigorous process to ensure its reliability and accuracy, including inter-rater reliability assessments. Details of its development, including inter-rater reliability assessment, have been published in a peer-reviewed journal and can be accessed for further information [14]. It consists of 12 subsections, and a summary of the questionnaire is attached as a supplementary file (Supplementary material 2).

### 2.6. Sample size

The sample size was calculated to detect a 10% mean score difference in total nutrition knowledge (TNK) measured by the SLn-SNKQ between the intervention and control groups after the intervention (at 16 weeks). The calculation was based on a 10% difference, as it represents a meaningful improvement in nutrition knowledge, supported by a prior study [23]. The sample size calculation incorporated effect size and variability, ensuring 80% power and a 95% confidence interval while accounting for a 20% dropout rate. This resulted in a required sample size of 30 participants. Participants were randomly and equally assigned to the intervention (*n* = 15) and control (*n* = 15) groups using a computer-generated random number sequence. The formula used for sample size calculation, along with a detailed description, has been published elsewhere [24].

### 2.7. Randomization

Participants were randomly assigned to the intervention or control group in a 1:1 ratio. Randomization was facilitated by a computer-generated sequence using the National Cancer Institute’s Randomization Tool [25]. An independent researcher who was not involved in participant recruitment produced the allocation sequence for assigning participants according to the corresponding randomly generated numbers. While the randomization process did not explicitly stratify participants based on their baseline SNK or GNK scores, post-randomization analysis revealed that the groups were very similar in these baseline scores, which occurred by chance rather than by design.

### 2.8. Allocation concealment

To ensure allocation concealment and prevent bias, sealed opaque envelopes indicating the participant’s allocation based on their recruitment number were prepared in advance by an independent researcher not involved in any part of the study. These envelopes were provided to the researcher responsible for participant enrollment at the time of recruitment. Each sealed envelope was opened only at the point of participant assignment, revealing the allocated group (intervention or control). This mechanism ensured that allocation remained concealed until the participant was ready to be assigned.

Eligible participants were informed of their randomized assignment only after completing screening and baseline assessments. The allocation sequence was generated using computer randomization by a team member who was not involved in participant recruitment.

### 2.9. Blinding

Outcome assessors and data analysts were blinded to intervention assignment to maintain single blinding, although participants were not blinded due to the nature of the intervention. The blinding procedure was maintained throughout the study, and unblinding, if required, would involve only outcome assessors and data analysts.

### 2.10. Statistical methods

The normality of GNK and SNK scores was assessed using the Shapiro-Wilk test prior to selecting appropriate statistical methods. As the data were normally distributed, parametric tests were employed to analyze continuous variables (e.g. mean GNK and SNK scores). For comparing the proportion of participants achieving a 10% or greater increase in SNK scores between the intervention and control groups, Fisher’s exact test was used, as it is appropriate for categorical (yes/no) data. To evaluate the effectiveness of the intervention, an independent samples t-test (two-tailed) to compare the post-intervention SNK scores between the two groups, and paired samples t-tests (two-tailed) were used to assess pre- to post-intervention changes within each group separately. All statistical analyses were conducted using SPSS (version 23), and significance was set at *p* < 0.05.

## 3. Results

Among the 30 participants initially enrolled in the trial, 13 in the IG and 14 in the CG completed the 16-week study (Figure 1). Results are reported for the 27 participants who completed the study, using the per-protocol analysis method. The mean age of the IG was 23.4 ± 2.8 years, with a gender distribution of 8 males and 5 females. The CG had a mean age of 21.9 ± 3.9 years, with 9 males and 5 females. The baseline demographic and anthropometric characteristics of the study participants are presented in Table 2. Regarding education, 38% of the IG and 42% of the CG had completed school education level I (10 years of school), while 46% of the IG and 42% of the CG had completed school level II (13 years of school). Sixteen percent of each group was enrolled in or had completed a degree or diploma. The sports experience of the IG recorded a mean of 7.15 ± 3.60 years, while the CG had a mean of 5.92 ± 3.73 years (*p* = 0.394). Sports experience refers to the total number of years the participants have been involved in organized sports, including any sport, not just track and field. Regarding performance levels, 38% of the IG and 36% of the CG were classified as elite athletes, while 62% of the IG and 64% of the CG were highly trained athletes. Participants were involved in four track and field categories: one sprinter in each group, 7 middle-distance runners in the IG and 10 in the CG, three long-distance runners in the IG and two in the CG, and two jumpers in the IG and one in the CG. At baseline, the total nutrition knowledge scores were comparable between the groups, with the IG scoring 24.96 ± 9.25 and the CG scoring 23.59 ± 4.01 (out of 75 marks) (*p* = 0.855).

**Table 2.** Baseline demographic and anthropometric values of study participants.

Variable	IG (*n* = 13) Mean ± SD	CG (*n* = 14) Mean ± SD	*p-*value
Age (y)	23.4 ± 2.8	21.9 ± 3.9	*p* = 0.260
Gender Male	8 (62%)	9 (64%)	*p* = 1.000
Female	5 (38%)	5 (36%)	
BMI (kg/m^2^)	19.37 ± 1.80	18.64 ± 1.73	*p =* 0.293
Education level: n (%)			*p =* 0.368
School education level I	5 (38%)	6 (42%)	
School education level II	6 (46%)	6 (42%)	
Completed/enrolled in a degree/diploma	2 (16%)	2 (16%)	
Sports experience (y)	7.15 ± 3.60	5.92 ± 3.73	*p* = 0.394
Level of performance: n (%) Elite	5 (38%)	5 (36%)	*p* = 0.394
Highly trained	8 (62%)	9 (64%)	
Main sport event: n (%)			*p =* 1.000
Sprinting	1 (7%)	1 (7%)	
Middle-distance running	7 (54%)	10 (71%)	
Long-distance running	3 (23%)	2 (14%)	
Jumping	2 (16%)	1 (8%)	
Total nutrition knowledge	24.96 ± 9.25	23.59 ± 4.01	*p* = 0.855

BMI: Body Mass Index, CG: Control Group, IG: Intervention Group, School education level I: 10 years of school education, School education level II: 13 years of school education.

**Figure 2.**
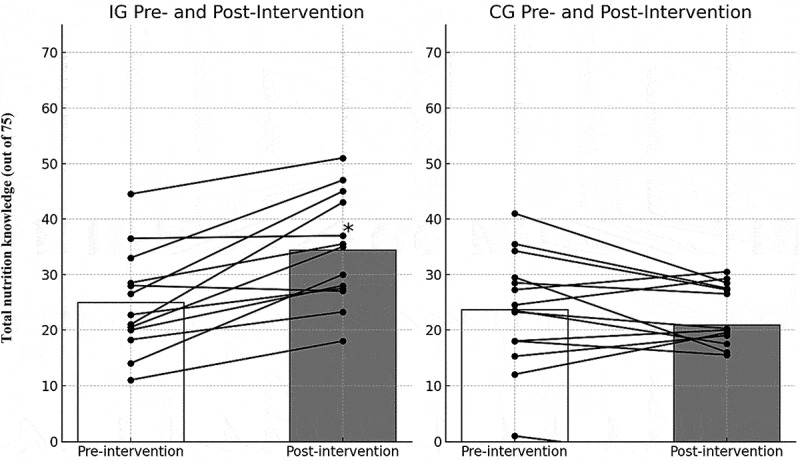
Change in total nutrition knowledge from pre- to post-intervention in the intervention and control groups.

Following the 16-week evidence-based sports nutrition education program, delivered through dual methods, a significant improvement in mean TNK was observed (IG: 34.41 ± 4.15 vs. CG: 20.96 ± 4.38; *p* = 0.01) (Figure 2), as well as in GNK (IG: 24.54 ± 3.66 vs. CG: 15.64 ± 2.33; *p* = 0.004) and SNK (IG: 9.87 ± 3.87 vs. CG: 5.32 ± 4.07; *p* = 0.006). Similarly, a significant improvement in the change in TNK was reported among participants in the IG following the 16-week sports nutrition intervention, compared to those in the CG (Table 3); (IG: 9.45 ± 0.10 vs. CG: −2.63 ± 0.37; *p* < 0.0001), GNK (IG: 4.47 ± 0.08 vs. CG: −1.28 ± 0.09; *p* < 0.0001), and SNK (IG: 4.99 ± 0.72 vs. CG: −1.25 ± 0.74; *p* < 0.0001) (Figure 2).

*=Statistically significant change (*p* < 0.05) with 95% confidence intervals of 1.84–4.01.

**Table 3.** Comparison of changes in each subsection, GNK, SNK, and total NK scores between the IG and CG.

Variable and the maximum score	IG (n=13) Mean ± SD				CG (n=14) Mean ± SD				p-value for IG vs CG			Effect Size (Cohen’s d)	95% CI
	Pre	Post	Change in NK	p-value	Pre	Post	Change in NK	p-value	Pre	Post	Change in NK		
Food types (15)	10.54 ± 1.39 (7–13)	10.62 ± 1.52 (8–15)	0.08±0.13; p=0.828	0.828	10.31 ± 5.78 (7–12)	7.72 ± 4.47 (7–13)	−2.59±1.31	0.272	0.855	0.160	<0.0001	0.62	(-0.15-1.40)
Macronutrients (5)	3.69 ± 1.29 (2–3)	3.92 ± 2.06 (3–4)	0.23±0.77; p=0.592	0.592	3.38 ± 2.36 (2–3)	3.21 ± 2.01 (2–3)	−0.17±0.35	1.000	0.374	0.374	0.105	0.21	(-0.55-0.97)
Micronutrients (5)	0.92 ± 1.50 (2–3)	2.85 ± 1.86 (3–4)	1.93±0.36; p=0.006	0.006	2.28 ± 1.86 (2–3)	2.50 ± 2.65 (2–3)	0.22±0.79	0.648	0.159	0.697	<0.0001	1.01	(0.21-1.81)
Energy balance (5)	0.46 ± 1.44 (2–3)	1.30 ± 1.55 (4-5)	0.84±0.11; p=0.311	0.311	1.20 ± 1.25 (2–3)	1.54 ±1.59 (2–3)	0.34±0.34	0.261	0.054	0.128	<0.0001	0.37	(-0.39-1.13)
Hydration (10)	4.46 ± 2.30 (5-6)	5.38± 2.18 (7-8)	0.92±0.12; p=0.008	0.008*	5.38 ± 2.18 (5-6)	4.57 ± 1.79 (5-6)	−0.81±0.39	0.225	0.227	0.227	<0.0001	0.77	(-0.01-1.56)
Training meals (5)	2.31 ± 1.80 (2–3)	3.00 ± 1.50 (4-5)	0.69±0.3; p=0.337	0.337	3.00 ± 2.68 (2–3)	3.29 ± 2.09 (2–3)	0.29±0.59	0.763	0.027	0.027*	0.037	0.17	(-0.58-0.93)
Sports supplements (5)	0.42 ± 0.71 (2–3)	3.60 ± 2.48 (4-5)	3.18±1.77; p=0.094	0.094	1.21 ± 0.25 (2–3)	0.46 ± 2.72 (2–3)	−0.75±2.47	0.175	0.004	0.004	<0.0001	7.50	(5.36-9.64)
Miscellaneous (5)	2.15 ± 1.86 (2–3)	2.46 ± 1.51 (4-5)	0.31±0.35; p=0.472	0.472	2.77 ± 2.13 (2–3)	2.57 ± 1.87 (2–3)	−0.20±0.26	0.351	0.867	0.867	<0.0001	0.25	(-0.50-1.01)
GNK (50)	20.07± 3.58 (15–25)	24.54± 3.66 (35–45)	4.47±0.08; p=0.004	0.004	16.92± 2.24 (10–25)	15.64±2.33 (10–25)	−1.28±0.09	0.650	0.004	0.004	<0.0001	1.94	(1.03-2.86)
SNK (25)	4.88±3.15 (10–15)	9.87 ± 3.87 (20–25)	4.99±0.72; p=0.002	0.002	6.57±3.33 (10–15)	5.32 ± 4.07 (10–15)	−1.25±0.74	0.423	0.006	0.006	<0.0001	1.92	(1.01-2.84)
Total (75)	24.96±4.25 (25–40)	34.41 ±4.15 (55–70)	9.45±0.10; p=0.004	0.004	23.59 ± 4.01 (20–40)	20.96±4.38 (20–40)	−2.63±0.37	0.137	0.855	0.001	<0.0001	2.93	(1.84-4.01)

Additionally, significant improvements in nutrition knowledge were observed across all subsections of the SLn-SNKQ in the IG compared to the CG (*p* < 0.0001), except the macronutrient section. Within the IG, knowledge of micronutrients (*p* = 0.006) and hydration (*p* = 0.008) showed statistically significant increases compared to baseline values. On the contrary, these measurements either decreased or remained unchanged in the CG, as shown in Table 3. Furthermore, when assessing the primary outcome, 10 out of 13 athletes (77%) in the IG achieved at least a 10% increase in SNK scores following the intervention, whereas in the CG, only 3 out of 14 athletes (21%) achieved this increase. Additionally, when evaluating TNK individually, 12 out of 13 athletes (92.3%) in the IG showed improvement, while only 5 out of 14 athletes (35.7%) in the CG demonstrated improvement, and 9 athletes had deteriorated SNK scores (Figure 2).

Based on the data presented in Table 4, the IG demonstrated a statistically significant increase in TNK from pre- to post-intervention (mean change = +9.45 ± 0.10, *p* = 0.004), while CG showed a non-significant decrease in knowledge (mean change = –2.63 ± 0.37, *p* = 0.137).

**Table 4.** Within-group changes in total nutrition knowledge from pre- to post-intervention in the intervention and control groups.

Group	Pre-Intervention TNK (Mean ± SD)	Post-Intervention TNK (Mean ± SD)	Change in TNK (Mean ± SD)	p-value	Effect Size (Cohen’s d)	95% CI
Intervention (IG)	24.96 ± 4.25	34.41 ± 4.15	+9.45 ± 0.10	.004	0.855	1.84–4.01
Control (CG)	23.59 ± 4.01	20.96 ± 4.38	−2.63 ± 0.37	.137	0.001	−0.74–0.74

TNK (Total Nutrition Knowledge) is measured on a scale from 0 to 75.

A statistically significant change (*p* < 0.05) is indicated by 95% confidence intervals.

Effect size (Cohen’s d) indicates the magnitude of the change within each group.

## 4. Discussion

To the best of our knowledge, this is the first study conducted in Sri Lanka to evaluate the effectiveness of a 16-week, evidence-based, culturally appropriate sports nutrition education program delivered through dual methods: face-to-face meetings and online support – in improving SNK among track and field athletes. Our hypothesis was that the IG would achieve a significantly higher proportion of participants with at least a 10% increase in mean TNK scores by the end of the 16th week compared to the CG. Consistent with our hypothesis, our findings demonstrated that the 16-week nutrition education intervention led to a significant improvement in TNK, with 77% of participants in the IG achieving at least a 10% increase in SNK, compared to only 21% in the CG.

The dual-method approach, combining in-person meetings and virtual support, was integral to the success of the intervention. The in-person sessions provided an opportunity for participants to engage directly with the content and the instructor, allowing for more personalized interaction and clarification of any questions or concerns. These face-to-face sessions fostered a sense of connection and accountability, which is crucial for building trust and enhancing engagement in educational programs. Participants were also given the opportunity to record the sessions, ensuring they could revisit the material as needed, which supported better retention of information.

On the other hand, the virtual component provided flexibility and convenience, allowing athletes to access study materials and participate in discussions at their own pace. We also provided many study materials for participants to refer to, ensuring they had ample resources to deepen their understanding of the topics covered. This was particularly important given the geographical dispersion of participants. Virtual follow-ups via WhatsApp and phone calls helped reinforce the material and allowed for continuous engagement beyond the in-person sessions. This combination of real-time, face-to-face interaction with flexible, on-demand virtual support addressed the diverse needs of the athletes, making the program more accessible and adaptable to their busy training schedules. The benefits of the dual-method approach are particularly relevant in the context of track and field athletes, who often face logistical challenges related to travel and time commitments. The in-person sessions allowed for deeper engagement, while the virtual component provided continuous access to support and resources, which is particularly valuable for athletes who may be unable to attend all in-person meetings due to travel or training schedules. This method not only increased the overall accessibility of the program but also ensured that athletes remained engaged throughout the intervention period, contributing to the positive outcomes observed in the IG. By blending in-person and virtual methods, the intervention maximized the benefits of both approaches. Face-to-face interactions facilitated personalized learning, while virtual components provided flexibility and ongoing support. This hybrid model offers a scalable solution for delivering nutrition education programs, especially in settings where logistical barriers may limit the feasibility of traditional, solely in-person interventions. Future research should explore the potential of dual-method interventions in other contexts and consider the relative effectiveness of in-person versus virtual methods for different types of educational content and target populations.

While the intervention showed overall improvements in SNK, specific areas of weakness within SNK remained notable. For example, knowledge related to micronutrient intake and hydration strategies demonstrated greater variability in improvement among the athletes. Similarly, while hydration strategies showed positive changes, applying this knowledge in real-life settings remained a challenge. These findings suggest that future interventions should focus on reinforcing these weak areas by integrating more practical, hands-on learning experiences to bridge the gap between theoretical knowledge and real-life application, especially in high-performance settings.

The current intervention included three individual in-person sessions and online support. In addition, we first provided athletes with a substantial understanding of GNK, covering broad topics such as macronutrients, micronutrients, energy balance, and hydration, using a specifically developed and well-validated guideline tailored for Sri Lankan track and field athletes [22]. This was followed by imparting knowledge specific to sports nutrition. Given that nutrition knowledge among track and field athletes in Sri Lanka is generally limited, this structured approach likely led to significant improvements in their nutrition knowledge. Our finding aligns with previous research showing an improvement in nutrition knowledge among highly trained UK adolescent swimmers following a seven-week, classroom-based nutrition education intervention after their regular swim training [15]. Similarly, several trials have reported results consistent with the current findings, demonstrating the effectiveness of nutrition education interventions for adolescent athletes across various sports, including dance [26], endurance sports [27], swimming [28], and soccer [29]. Furthermore, additional studies have reported gains in sports nutrition knowledge, though direct comparisons to our findings are challenging due to variations in the nature and structure of the sports nutrition interventions employed [30–33]. Our study utilized the SLn-SNKQ, a validated tool with robust psychometric properties, providing a reliable means to assess SNK among Sri Lankan track and field athletes [14]. The novelty in the current study was the dual methods approach used to enhance the nutrition knowledge of track and field athletes in both elite and highly trained categories. Given the observed improvements in nutrition knowledge and the logistical challenges of athletes being in various regions, practitioners may consider implementing a similar dual-method nutrition education intervention, as employed in our study, to achieve comparable positive outcomes among athletes.

Our study’s novelty lies in its culturally sensitive approach and dual-method delivery, which was designed to account for logistical challenges such as athletes’ geographical distribution and varying educational backgrounds. The personalized nature of the intervention, with opportunities for follow-up questions via WhatsApp and voice recordings, ensured that the learning experience was more accessible and flexible. This method also minimized the risk of contamination between the IG and CG, further strengthening the validity of our findings.

Moreover, while the intervention was effective in improving knowledge, it is important to consider how these gains might translate into tangible improvements in athletic performance and dietary habits. The direct link between improved SNK and performance outcomes remains an area for further investigation. Future studies should aim to assess not only the retention of knowledge but also whether athletes can apply this knowledge to enhance their training, recovery, and overall performance. Additionally, given the significant improvements in dietary knowledge, it would be valuable to explore whether these changes were reflected in actual dietary behaviors and whether these behaviors lead to improved physical outcomes such as body composition, energy levels, or reduced injury rates.

One of the key strengths of the current intervention was the presence of a CG that was comparable to the IG regarding all known and unknown confounders, a feature that is lacking in some similar studies [15,17]. Furthermore, unlike other studies [15,17] that focused solely on SNK, we devoted equal attention to both GNK and SNK to achieve optimal results. The duration of each face-to-face sports nutrition consultation was sufficient for participants to ask questions, and they were permitted to voice record each meeting with the principal investigator (PI) using their mobile phones, allowing them to review the sessions repeatedly. To enhance clarity, study materials were delivered in participants’ native languages and numerous opportunities were provided for them to ask questions via WhatsApp messages and phone calls. Furthermore, the risk of contamination of the nutrition knowledge intervention between the IG and CG was minimized due to the personalized nature of the sessions, and follow-ups were conducted through direct WhatsApp messages and telephonic conversations throughout the 16 weeks. Participants in the CG were reassured that they would receive a one-on-one session with the PI and all study materials at the end of the study. Moreover, considering the athletic disciplines of the study participants, the majority were from the middle-distance running category, which is the predominant track and field event in Sri Lanka. This enhances the generalizability of the study findings to a large cohort of this athletic population in Sri Lanka.

One of the main limitations of our study is that it focused solely on track and field athletes, which may limit the generalizability of the findings to athletes from other sports. However, the questions related to GNK and SNK are relevant to athletes in various sports. By including the maximum possible scores for each section of the SLn-SNKQ, it becomes evident where the participants’ strengths and weaknesses lie in their nutrition knowledge. Additionally, reporting the percentage of correct responses for each section allows for a more nuanced understanding of the effectiveness of the intervention in specific areas of sports nutrition knowledge.

Although macronutrient knowledge showed some improvement, the gains were comparatively smaller, likely reflecting participants’ existing baseline understanding in this core area. This suggests that future nutrition education programs could benefit from incorporating more advanced or practical content on macronutrients to enhance athletes’ comprehension and application. Furthermore, certain areas such as hydration and micronutrients continued to pose challenges for participants, highlighting the need for ongoing emphasis on these topics in future interventions. It is also important to acknowledge that this culturally specific nutritional intervention may not be fully generalizable to countries with different dietary patterns. On the other hand, although the total nutrition knowledge scores improved significantly in the intervention group, the overall post-intervention mean remained below 50%. This finding should be interpreted in light of the validated assessment tool used, which comprehensively evaluates both general and sports-specific nutrition knowledge across multiple domains and is considered challenging even for individuals with advanced nutrition training. Given the short duration of the intervention (16 weeks), these results suggest that while the program was effective in initiating improvements, longer-term and more intensive nutrition education and support may be necessary to achieve more substantial gains in knowledge, as well as to evaluate the durability of these improvements and their potential impact on long-term health and performance.

In conclusion, the results of this study suggest that a 16-week dual-method sports nutrition education intervention can significantly enhance the SNK of track and field athletes in Sri Lanka. The intervention employed in the present study may serve as a valuable framework for sports nutrition practitioners aiming to improve SNK among athletes, particularly within the context of culturally appropriate education. However, future studies should evaluate the impact of improvements in nutrition knowledge on actual dietary intakes and whether these changes contribute to better performance outcomes and overall well-being. Further research is also warranted to investigate additional interactive methods for delivering nutrition education interventions, such as online workshops, group discussions, and the use of mobile applications or social media platforms, over an extended period.

## Supplementary Material

Supplemental Material

## Abbreviations

BMI: Body Mass Index
CG: Control Group
GNK: General Nutrition Knowledge
IG: Intervention Group
NK: Nutrition Knowledge
PI: Principal Investigator
SLn-SNKQ: Sri Lankan Sports Nutrition Knowledge Questionnaire
SNK: Sports Nutrition Knowledge
SNKQ: Sports Nutrition Knowledge Questionnaire
TNK: Total Nutrition Knowledge
